# Pharmacokinetic Parameters Determination and *In Vitro–In Vivo* Correlation of Ileocolonic-Targeted pH-Responsive Coated Mini-Tablets of Naproxen

**DOI:** 10.3797/scipharm.1503-16

**Published:** 2015-06-02

**Authors:** Mohd Abdul Hadi, Nidagurthi Guggilla Raghavendra Rao, Avanapu Srinivasa Rao

**Affiliations:** 1 Department of Pharmaceutics, Bhaskar Pharmacy College, Yenkapally (V), Moinabad (M), R. R. District, Hyderabad-500075, Telangana, India; 2 Department of Pharmaceutics, Sree Chaitanya Institute of Pharmaceutical Sciences, LMD Colony, Thimmapur, Karimnagar-505001, Telangana, India; 3 Bhaskar Pharmacy College, Yenkapally (V), Moinabad (M), R. R. District, Hyderabad-500075, Telangana, India

**Keywords:** Coated-mini-tablets, Naproxen, Eudragit L100, Eudragit S100, Rabbits

## Abstract

This research work aims to determine the pharmacokinetic parameters and *in vitro-in vivo* correlation of the selected ileocolonic-targeted coated mini-tablet filled capsule formulation of naproxen. The pure suspension and coated mini-tablet filled capsule formulation of naproxen were administered to adult albino rabbits through the oral route. The samples were analyzed for naproxen by an HPLC method. For the pure drug suspension, the peak plasma concentration was found as 8.499±0.029 μg/ml at 1.139±0.010 hours and the half-life was found to be 9.459±0.387 hours, whereas for the formulation the peak plasma concentration was found as 6.814±0.037 μg/ml at 8.042±0.069 hours and the half-life was found to be 19.657±0.359 hours. This decreased the peak plasma concentration at a delayed time and increased the half-life of the capsule formulation in comparison with the pure drug suspension which showed that naproxen was only targeted to the ileocolonic region. A significant *in vitro-in vivo* correlation (i.e. R^2^=0.9901) was also obtained. Thus, the results of these findings suggest that naproxen formulated as coated mini-tablets can be suitable for targeted ileocolonic drug delivery.

## Introduction

Rheumatoid arthritis is a chronic inflammatory syndrome which causes the destruction of joint integrity. Patients with this disease have joint pain and functional disability symptoms which mainly persist in the early morning hours [1, 2]. These symptoms occur due to diurnal variations in the levels of circulating proinflammatory cytokines, interleukin-6, and/or tumor necrosis factor-α [3]. The chronotherapy concept can be used for better treatment of rheumatoid arthritis so that the highest amount of drug can be maintained in the bloodstream during the early morning [4, 5]. In this instance, colon targeting of a drug or intentionally delayed absorption can be preferable to have a uniform therapeutic effect. The drug can be delivered in a higher amount during its greatest need, because the release of the drug occurs after a lag time. Thus, the peak pain and stiffness symptoms of the disease can be overcome and good patient compliance can be achieved [1].

Dew *et al*. developed the first colonic-targeted pH-responsive drug delivery system and it is most specifically referred to as ‘ileocolonic-targeted drug delivery’ rather than a colonic-targeted drug delivery system [6–9].

A number of approaches can be used for targeting the drugs at the colonic junction. Some of them are by using enzyme- and pH-dependent approaches [10]. In the enzyme-dependent approach, it makes use of such carriers or polymers which are degraded by enzymes produced by the colonic bacteria, whereas in the pH-dependent approach, it depends upon the increased pH of the gastrointestinal tract, i.e. from the stomach (pH 1.5–3) to terminal ileum (pH 7–8) [11].

Controlling the size and size distribution of the pellets is more problematic prior to the coating process. So, in order to achieve a reproducible release profile, both a desirable narrow particle size distribution and pellet size are important, which are not easy to achieve. Granules have irregular shapes and high porosity, thus making coating difficult [12].

Mini-tablets are very small tablets whose diameter is equal to or smaller than 3 mm, which can be placed in sachets or filled into a capsule shell for easy administration. Moreover, they are more uniform in size, and therefore, much less unit-to-unit variation in the drug occurs and accurately weighed amounts of drugs can be loaded into the mini-tablets. They are very easy to manufacture and can even be coated so as to delay the drug release due to excellent smooth surface area. Thus, they can be considered as good substitutes for pellets and granules [13]. In the present work, the reason for designing coated mini-tablets in a filled capsule formulation is to develop a more reliable dosage form which possesses all the advantages of a larger single-unit tablet, yet avoid the problems such as the danger of dose dumping and alteration in the release profile of a drug due to unit-to-unit variation.

Naproxen is a derivative of naphthylpropionic acid which belongs to the class of NSAIDs and it has been found to be effective in both experimental and clinical pain for rheumatoid arthritis. By keeping in view the need to target naproxen during the time of its greatest need, a novel pH-responsive coated mini-tablet filled capsule system was developed in our previous study [12]. In that study, the best formulation was selected based on the *in vitro* results. It was identified as the best due to its more targeted release in the ileocolonic pH buffer (i.e. 7.2 phosphate buffer). These optimized mini-tablets were prepared by the direct compression method and were then coated with a 1:2 ratio of Eudragit L100: Eudragit S100 with 20% coating level. In this present research work, the aim was to evaluate the *in vivo* performance of the selected ileocolonic-targeted coated mini-tablet filled capsule formulation of naproxen. The investigation involved the evaluation of pharmacokinetic parameters of naproxen using albino rabbits as the experimental animals.

## Materials and Methods

### Materials

Naproxen was obtained as a gift sample from IPS Pharma Training Institute, Hyderabad, India. The pH-sensitive polymers (Eudragit® L-100 and Eudragit® S-100) were obtained as gift samples from Degussa India Pvt. Ltd., Mumbai, India. Sodium alginate, guar gum, microcrystalline cellulose Avicel PH102, and Aerosil^®^ were purchased from SD Fine Chemicals, Mumbai, India. Magnesium stearate was purchased from Himedia Chem Lab, Mumbai, India. Empty HPMC capsules of almost all sizes were obtained as gift samples from ACG Associated Capsules Pvt. Ltd. Mumbai, India. All the solvents which were used in the analysis of HPLC were of HPLC grade. The remaining materials used were of analytical grade.

### Methods

#### Experimental Design

The protocol of the above experiment was approved by the Institutional Animal Ethics Committee (IAEC) of the Luqman College of Pharmacy, Gulbarga (No: LCP/PhD.IAEC/Jan-2014/10) and all the animals were maintained as per the guidelines of CPCSEA. In this study design for the assessment of pharmacokinetics, six albino rabbits of either sex weighing 2 kg were selected and divided into two groups of three animals each. All of the experimental animals were housed individually in a loose box and were given a balanced ration twice daily and water ad libitum. The first group of animals received the optimized coated mini-tablet filled capsule formulation equivalent to 16.666 mg of naproxen through the oral route by gavage, whereas the second group of animals received 16.666 mg of a 4.1665 mg/ml naproxen suspension through the oral route by gavage [14].

#### Procedure for the Preparation of the Optimized Coated Mini-Tablet Filled Capsule Formulation

Core mini-tablets of naproxen were prepared by the direct compression method using 3 mm round concave punches in a rotary tablet press (Model RSB-4, Rimek Mini-Press, Karnavathi Engineering, Ahmedabad). The composition of the core mini-tablets is shown in Table 1. A sufficient quantity of naproxen core mini-tablets along with the placebo tablets were then coated with 500 ml of coating solution as shown in Table 2 up to the 20% coating level in a ‘6’–inch lab-scale coating pan using a spray gun (supplied by United Technologies Company, Mumbai, India). The coating solution was prepared by first adding the combination of Eudragit polymers in 50% of the solvents and stirring them completely for a period of approximately 60 minutes till both the polymers completely dissolved. Secondly, after adding triethyl citrate and talc in the remaining solvent mixture, it was stirred with a high speed mixer for 10 minutes. Then this solution of triethyl citrate and talc was poured slowly into the polymer solution in the stirring motion and finally passed through a 0.5 mm sieve. The operating parameters were: tablets charge 100 g; preheating temperature 40 ± 5°C; preheating time 20 min; inlet air temperature 45 ± 5°C; nozzle diameter 1.0 mm; atomization pressure 2.0 bar; spray rate 8–10 ml/min. In our previous study [12], the optimized coated mini-tablets filled capsule formulation was developed by filling 15 coated mini-tablets (equivalent to 250 mg of naproxen) in an empty size ‘0’ HPMC capsule. For preparing the capsule formulation, in order to be easily administered to the rabbits of this study, one coated mini-tablet (equivalent to 16.666 mg) of naproxen was filled into a size ‘4’ HPMC capsule.

**Tab. 1:**
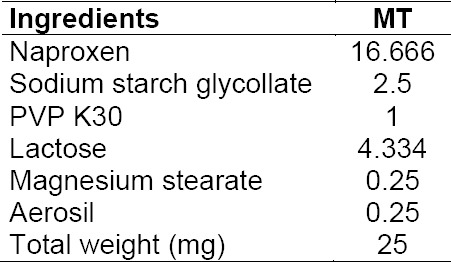
Composition of the core mini-tablet

**Tab. 2:**
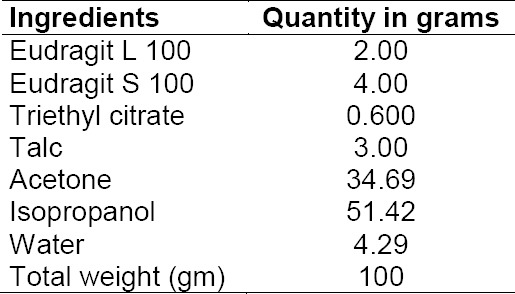
Composition of the coating solution

#### In Vitro Dissolution Test

In-vitro dissolution studies were performed by dipping one capsule filled with 15 coated mini-tablets at a time in a basket type USP-XXIII dissolution test apparatus. When conducting the studies, 750 ml of 1.2 pH media were first used for 2 hours, and then replaced with the 900 ml of fresh 6.5 pH phosphate buffer. After 1 hour, the media was again replaced with 900 ml of fresh 6.8 pH phosphate buffer and after 2 hours was further replaced with 900 ml of fresh 7.2 pH phosphate buffer for a period of 3 hours. Rotation speed and temperature were set at 100 rpm and 37 ± 0.5°C, respectively. At fixed time intervals, 5 ml of dissolution media was withdrawn and then replaced with fresh dissolution media. These withdrawn samples were analyzed by UV absorption spectroscopy at 230 nm, 329 nm, 329 nm, and 331 nm for 1.2, 6.5, 6.8, and 7.2 pH buffers, respectively [12].

#### Assessment of Pharmacokinetic Data and Conditions Used in Chromatography

After the administration of the coated mini-tablet filled capsule formulation and pure suspension of naproxen in the first and second groups, respectively, blood samples (200 μL) were withdrawn from the marginal ear vein of rabbits at scheduled intervals of time (0, 0.5, 1, 1.5, 2, 4, 6, 8, 10, 12, and 24 hours) and collected into heparinized tubes. From the samples of blood, the plasma was obtained using centrifugation for a period of 10 min, which was then kept in glass tubes and frozen at −25°C ± 2.0. Before analyzing using HPLC, a 100 μL solution of an internal standard i.e. indomethacin (5 μg/ml) and 10 μL of methanol were added to 100 μL of plasma aliquots and were mixed for 5 sec in a vortex mixer. All the sample tubes were centrifuged at 8000 rpm for 10 min, and after transferring the organic layer to a new glass tube, they were evaporated to dryness using a nitrogen stream at 40°C. After reconstituting the residue with 500 μL of the mobile phase (which was a mixture of potassium dihydrogen phosphate and methanol in a 60:40 ratio), it was filtered and an aliquot of 20 μL was injected into the HPLC system. The HPLC system (Waters W2695 Separation Module) consisted of an HPLC pump (W2690/5) and autosampler with a PDA detector, model (2998). Chromatographic separation was carried out using an ACE 5CN column (4.6 × 250 mm, particle size: 5 microns) at a wavelength of 266 nm. The flow rate was 1 mL/min [14].

#### Pharmacokinetic Analysis

For the pure suspension and coated mini-tablet filled capsule formulation of naproxen, the pharmacokinetic parameters such as the maximum plasma concentration of the drug (Cmax, μg/ml) and time taken to reach the maximum plasma concentration of drug (Tmax, hr) were determined from the plasma concentration versus time data. The elimination rate constant (Kel, hr^−1^) was calculated by the least-squares regression analysis technique. The elimination half-life (t1/2e, hr) was calculated from the equation: t½ = 0.693/Kel. The absorption rate constant (Ka, hr^−1^) and absorption half-life (t1/2a, hr) were determined by using the method of residuals. The area under the plasma concentration versus time curve (AUC(0-t), μg.h/ml) and the area under the first moment curve (AUMC(0-t), μg.h/ml) from 0 to 24 hours were calculated from the linear trapezoidal method. The area under the plasma concentration versus time curve was (AUC(0-α), μg.h/ml), the area under the first moment curve was (AUMC(0-α), μg.h/ml), the mean residence time was (MRT(0-α), hr) from 0 to infinity, and the volume of distribution (V_d_, L), clearance (Cl, L.hr^−1^), and the relative bioavailability (RB) were calculated using the below-mentioned equations 1, 2, 3, 4, 5, and 6, respectively [15–19].


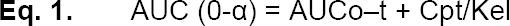



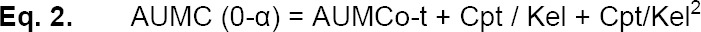



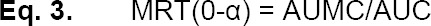



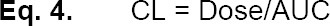



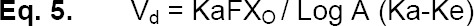



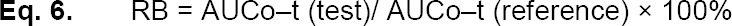


Where Cpt = Last plasma drug concentration observed at time t

Kel = Elimination rate constant

F = Fraction of drug absorbed

X_O_ = Dose administered

Log A = Y-intercept of the terminal portion of the plot (i.e log extrapolated plasma drug concentration versus time)

AUCo–t (test) = Area under plasma concentration versus time curve from 0 to 24 hours of the coated mini-tablet filled capsule formulation of naproxen

AUCo–t (reference) = Area under plasma concentration versus time curve from 0 to 24 hours of pure suspension of naproxen

#### In Vitro and In Vivo Correlation (IVIVC)

There are four types of modeling methods out of which level A is considered as the most widely used. In this method, the typical mathematical process involves the assessment of the percent cumulative drug released from the *in vitro* dissolution studies and considers the deconvolution of the *in vivo* plasma profile by the Wagner-Nelson method. This deconvolution process is performed to estimate the percent *in vivo* drug absorbed from the cumulative AUC. The equation used for calculating the percentage of naproxen dose absorbed is mentioned below:


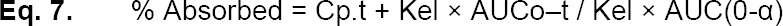


This is followed by comparing the *in vivo* fraction of drug released (FDR) to the *in vivo* fraction of the drug absorbed (FDA). Further, a linear correlation was established between the FDR and FDA by plotting the FDR on the x-axis and FDA on the y-axis. Then for this plot, the R^2^ value was determined [20–24].

#### Statistical Analysis

The pharmacokinetic parameters of naproxen which were calculated following the administration of both the treatments in rabbits were statistically evaluated using a paired t-test. The difference between the two related pharmacokinetic parameters were considered to be statistically significant at p < 0.05.

## Results

### In Vitro Dissolution Studies

**Fig. 1:**
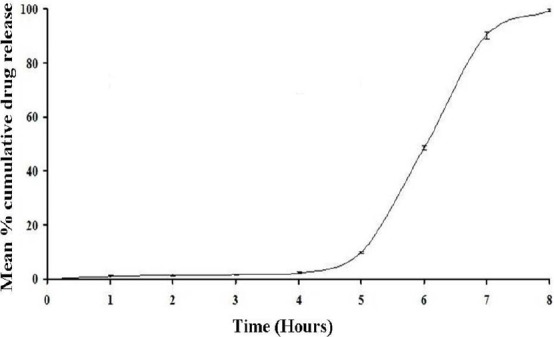
*In vitro* dissolution profile of the optimized coated mini-tablet filled capsule formulation of naproxen (mean ± SD; n=3)

From the in vitro dissolution testing, which was carried out in our previous study [12], it was found that the formulation prepared with a 1:2 ratio of Eudragit-L100 : Eudragit-S100 with a 20% coating level released naproxen after a lag time of 5.02 ± 0.40 hours and 99.40 ± 0.40% at the end of 8 hours as represented in Figure 1. This formulation was found to be the most suitable because it met the desired criteria.

**Tab. 3:**
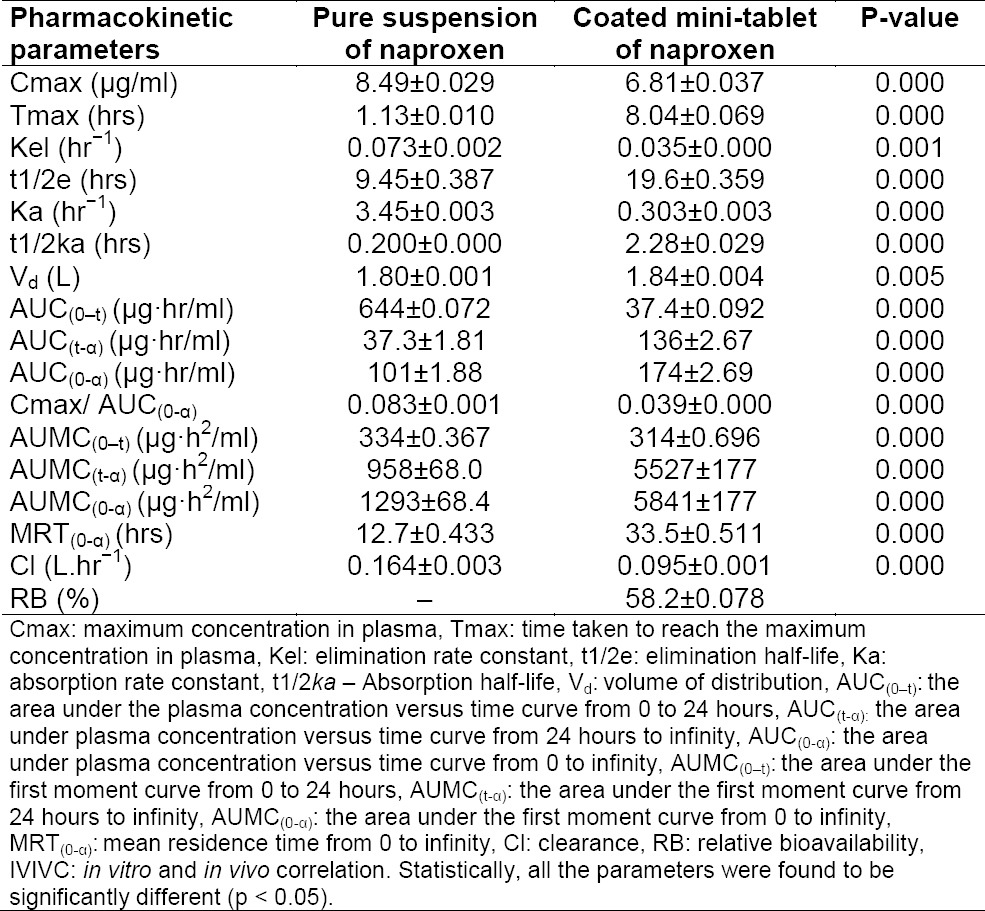
Mean pharmacokinetic parameters of naproxen after administration of two different treatments

### In Vivo Study and Pharmacokinetic Analysis

**Fig. 2:**
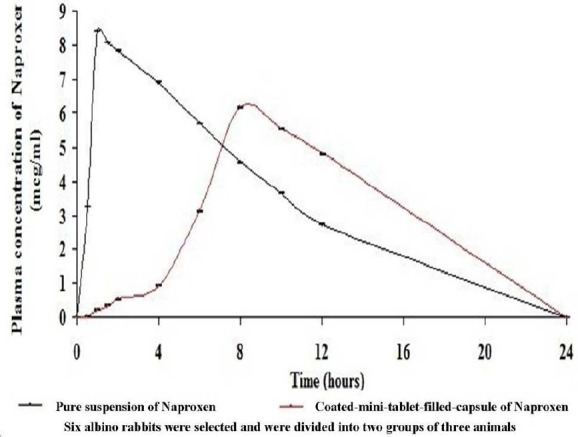
Mean plasma concentrations level versus time profile curve of naproxen after administration of 16.666 mg of the oral dose for both the treatments (mean ± SD; p < 0.05; n=3)

The mean pharmacokinetic parameters and the plasma concentrations versus time profile curve following oral administration of 16.666 mg of naproxen for the (i) pure drug suspension and (ii) optimized coated mini-tablet into three rabbits for each batch are shown in Table 2 and graphically represented in Figure 2, respectively. It was found that for the coated mini-tablet filled capsule formulation, the C_max_ was decreased at a delayed T_max_ in comparison with the pure drug suspension. Moreover, the curve also shows that in the initial hours, the plasma concentration of naproxen was less. The relative bioavailability was found to be high. Thus, it confirms that the highest concentration of naproxen was only targeted to the ileocolonic region.

### In Vitro-In Vivo Correlation

**Fig. 3:**
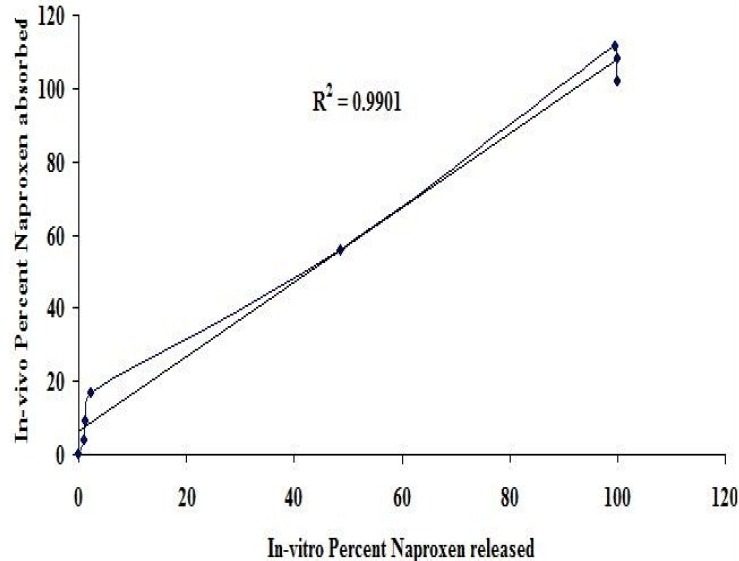
*In vitro* percent of naproxen released versus *in vivo* percent naproxen absorbed

A significant *in vitro/in vivo* correlation was also obtained with a high R^2^ value of 0.9901 and is graphically represented in Figure 3.

## Discussion

In the present pharmacokinetic studies of the coated mini-tablet filled capsule formulation, the administered dose of naproxen was not calculated as per the body weight or surface area of rabbits. Instead, the dose (16.666 mg) which is present in the formula of the individual coated mini-tablet of the optimized formulation was administered to the three animals of each group. The aim of this work was only to assess the pharmacokinetic parameters. Moreover, if the dose had been administered according to the body weight or surface area of the rabbits (which is 23.33 mg after converting 70 kg human dose, i.e. 250 mg to 2 kg rabbit dose by applying a conversion factor) [26], then it would have required fracturing a portion of the mini-tablet or varying the amount of the drug in the individual mini-tablet which may affect the drug-polymer ratio, coating film, weight, composition, or formula of the optimized coated mini-tablet formulation. These factors may cause a difference in pharmacokinetic parameters or may even result in a poor *in vitro-in vivo* correlation. Also, the most important advantage of the mini-tablets as a multiple unit drug delivery system is that they have accurate shapes, sizes, weights, density, and uniformity in drug content within the formulation. It means a single mini-tablet can be easily ingested by a rabbit and be used to assess the pharmacokinetic performance of the entire mini-tablet filled capsule formulation. In order to prevent deviation in the pharmacokinetic parameters of the rabbits, special care was also given to maintain the uniformity in weights of rabbits (i.e. 2 kg).

### In Vitro Dissolution Studies

The objective in the dissolution testing of our previous study [12] was to identify a suitable ratio and coating level of enteric polymers which releases naproxen after a lag time (<10%) of at least 5 hours or at the ileocolonic junction and maximum portion of naproxen till the end of 8 hours. Based on this, a suitable formulation should provide adequate lag time in pH 1.2, 6.5, and 6.8 dissolution media, but immediately release naproxen in pH 7.2. This assumption is based on the criteria that if the optimized formulation will be taken at night (i.e. before going to bed at 10:00 P.M), then it starts releasing naproxen after a lag time of 5 hours (i.e. at 03:00 A.M), thereby completely releasing naproxen at 06:00 A.M. This criterion was selected to target the maximum naproxen amount during early morning hours when the symptoms of rheumatoid arthritis will be at its peak.

In our previous dissolution studies, it was found that coating Eudragit L100 alone could not individually target the naproxen release in a pH 7.2 phosphate buffer because of its good solubility in a pH 6 phosphate buffer. But when core mini-tablets were coated with Eudragit S100 polymer alone, a complete lag time of 5 hours was easily obtained, but failed to release the maximum portion of naproxen till the end of 8 hours. Its release was still sustained after 8 hours. In order to adjust the release profile so that it should match the desired criteria, a mixture of both Eudragit polymers were coated on core mini-tablets. So, a series of trials with a different ratio combination and coating level of polymers were evaluated. Finally, the core mini-tablets when coated with a 1:2 ratio of Eudragit L100 : Eudragit S100 polymers with a 20% coating level met the desired criteria. Thus, this formulation was selected for pharmacokinetic studies in rabbits.

### In Vivo Study and Pharmacokinetic Analysis

In the present research work, the aim was to investigate the pharmacokinetic parameters of the coated mini-tablet filled capsule formulation of naproxen as a new dosage form and also to estimate the percentage of relative bioavailability.

From the results of the pharmacokinetic analysis, a clear remarkable difference was found between the two treatments. For the coated mini-tablet filled capsule formulation, the C_max_ was decreased at increased T_max_ when compared to the pure suspension of naproxen. Moreover, the curve shows that in the initial hours, the plasma concentration of naproxen was less. This is due to the reason that the presence of the Eudragit L100 and Eudragit S100 polymers’ coating in mini-tablets has pH-dependent solubility which retards the release of naproxen in the stomach and small intestine of rabbits. The release of naproxen was increased in the ileocolonic region of rabbits and thus has shown a high plasma concentration of naproxen which is due to the high solubility of Eudragit polymers in the ileocolonic pH. This decreased C_max_ at delayed T_max_ and lower plasma concentration of naproxen in the initial hours from the ileocolonic-targeted coated mini-tablet filled capsule formulation in comparison with the pure drug suspension shows that the naproxen was only targeted to the ileocolonic region and was not released in either the stomach or small intestine.

The coated mini-tablet filled capsule formulation was also characterized by a decrease in the elimination rate constant and absorption rate constant and an increase in the elimination half-life compared to the pure suspension of naproxen. The results clearly showed a flip-flop phenomenon, which has been mentioned in the literature [25], that is often associated with sustained or delayed release formulations. Another indication of delayed delivery of naproxen from the coated mini-tablet filled capsule formulation is a slower rate of absorption Cmax/AUC_(0-α)_ and a higher mean residence time compared to the pure suspension of naproxen. The volume of distribution was higher and clearance was also slower from the coated mini tablet-filled capsule formulation, indicating that the increase in mean residence time was due to delayed absorption of naproxen.

Moreover, the relative bioavailability was also found to be high and thus it indicates that naproxen in the coated mini-tablet formulation was absorbed into the blood circulation with a reasonable plasma concentration.

### In Vitro-In Vivo Correlation

When an *in vitro-in vivo* correlation was established by plotting the FDR on the x-axis and FDA on the y-axis, a correlation curve was obtained. For assessing the correlation value of this curve further, a straight trend line was added above it which intersected at three data points of the correlation curve giving a high R^2^ value, i.e. 0.9901. Thus, it indicates that an appreciable linear relationship was obtained between the naproxen fraction released and the naproxen fraction absorbed.

From the above results of pharmacokinetic parameters, it clearly suggests that the formula used to prepare the coated mini-tablets can be suitable for the ileocolonic-targeted delivery of naproxen and thus in the chronotherapeutic treatment of rheumatoid arthritis.

## Conclusion

The optimized coated mini-tablet filled capsule formulation of naproxen was efficiently evaluated for its pharmacokinetic parameters and *in vitro-in vivo* correlation. The results of the pharmacokinetic findings clearly suggest that naproxen administered as coated mini-tablet filled in a capsule may present a novel dosage form for ileocolonic-targeted delivery and thus in the chronotherapeutic treatment of rheumatoid arthritis. The results of the *in vitro-in vivo* correlation indicate an appreciable linear relationship between the naproxen fraction released and the naproxen fraction absorbed.
